# Current Trends, Spatio‐Temporal Dynamics of Microplastics Research and Global Public Health: A Scientometric Study

**DOI:** 10.1002/hsr2.70318

**Published:** 2025-01-08

**Authors:** Juan Alvitez, Luis Huarachi, Abigail Temoche, Carlos Medina, Daniel Alvitez‐Temoche, Miguel Cabanillas‐Lazo, Frank Mayta‐Tovalino

**Affiliations:** ^1^ Academic Department, Human Medicine Career Universidad Nacional Federico Villarreal Lima Peru; ^2^ Academic Department, Research, Innovation and Entrepreneurship Unit Universidad Nacional Federico Villarreal Lima Peru; ^3^ Academic Department Universidad de Huánuco Huánuco Perú; ^4^ Vicerrectorado de Investigación Universidad San Ignacio de Loyola Lima Peru

**Keywords:** bibliometrics, global public health, microplastics

## Abstract

**Purpose:**

To evaluate the current trends and spatiotemporal dynamics of microplastic research and public health through a scientometric study from 2019 to 2024.

**Methods:**

An observational and descriptive approach was applied to the published literature on microplastics and public health published in January 2019 and July 2024. A quantitative approach was used to analyze document production, author–country collaborations and thematic evolution. A comprehensive search of the Scopus database was conducted using a combination of keywords related to “microplastics” and “public health.” Papers that explicitly addressed these topics were considered for analysis. SciVal and R studio software were used for the analyses.

**Results:**

Significant growth in paper production was observed, with an increase in international collaboration and a diversity of paper types. The most prominent journals in this field include “Science of the Total Environment” and “Journal of Hazardous Materials”, which lead in terms of academic output and impact. There was an upward trend in the number of publications in the Q1 quartile, reflecting growing interest in this field. Several authors have demonstrated a significant impact on microplastic research and its impact on public health, highlighting the importance of further research in this field. In addition, 156 sources and 259 papers related to microplastics and public health research were identified, reflecting the diversity and richness of research in this field. The thematic evolution showed interesting changes in focus topics over time, highlighting the intersection of microplastics with topics such as “covid‐19” and “public health.”

**Conclusions:**

These findings underscore the relevance and urgency of microplastic and public health research and suggest promising directions for future research. The increasing volume of published research in these fields over time reflects the continuing importance of microplastics research and its impact on public health.

## 1 Introduction

Plastics in the environment break down into smaller fragments, less than 5 mm in size, known as secondary microplastics. This fragmentation occurs due to slow degradation processes such as photo‐ and thermo‐oxidation and, to a lesser extent, biodegradation. In contrast, primary microplastics are intentionally manufactured to be this size for use in products like cosmetics, toothpaste, and exfoliant, as well as in industrial applications like air blasting [1, 2].

Microplastics are a diverse class of pollutants that vary in size, shape (including spheres, fragments, and fibers), and composition. They include polymeric materials and a mixture of chemicals such as residual monomers, additives, and hydrophobic contaminants for environmental protection. Additionally, biofilms that form on microplastics can harbor harmful microorganisms [3].

The impact of microplastics on wildlife and ecosystems is increasing due to their widespread presence in the environment. They have been detected in seawater at concentrations of up to 102,000 particles per cubic meter and are also contaminating freshwater sources [1, 2, 3, 4]. Humans are exposed to microplastics through ingestion, dermal contact, or inhalation, which can lead to metabolic, circulatory, and immune system effects, as well as neurodegenerative diseases due to their neurotoxicity. This represents a major global public health concern [1, 4, 5].

Scientometric, which are quantitative methods used to study the development of science as an informational process, can be applied to analyze the bibliographic characteristics of scientific literature in a specific field, such as medicine. This analysis includes examining publications according to various variables of interest [6]. Key metrics, such as the number of citations and publications by different authors, research groups, and institutions over a certain period, are considered [7]. The resulting information is valuable for planning and managing economic and institutional resources directed toward research because it reveals the impact of scientific activity on society and its performance in the scientific community. Microplastics (MPs), have raised significant environmental concerns in aquatic ecosystems. However, there has been comparatively less research on their presence and impact in indoor environments, where people spend the majority of their time [8]. Conversely, it is crucial to focus on sustainable agricultural practices and efficient soil remediation methods to reduce particulate matter contamination. This approach supports environmental conservation, ensures food security, and safeguards human health [9]. Microplastics not only affect aquatic and terrestrial ecosystems, but also pose a direct threat to human health. Their presence in drinking water, food and the air we breathe can lead to adverse health effects, such as metabolic, immunological and neurodegenerative problems. In addition, the accumulation of microplastics in the environment can affect biodiversity and the quality of natural resources, which in turn impacts food security and the well‐being of communities [6, 7, 8, 9, 10].

Therefore, the purpose of this research was to conduct a scientometric analysis of the current trends and spatiotemporal dynamics of research on microplastics and public health, with the aim of evaluating its scientific impact in this field.

## 2 Methods

### 2.1 Study Design

This scientometric study applied an observational and descriptive approach to the published literature on microplastics and public health from 2019 to 2024. A quantitative approach was used to analyze document production, author–country collaborations and thematic evolution.

### 2.2 Search Strategy

On July 17, 2024, a comprehensive search of the Scopus database was conducted using a combination of keywords related to “microplastics” and “public health.” The search was limited to papers published between January 2019 and July 2024. The following formula was applied: TITLE‐ABS (“microplastics” OR “micro‐particles” OR “nano‐plastics” OR “microplastic particles” OR “plastic microbeads” OR “plastic fragments” OR “micro‐sized plastics” OR “tiny plastic particles” OR “microscopic plastics” OR “plastic fibers” OR “plastic debris” OR “nano plastics” OR “plastic pollutants”) AND TITLE‐ ABS (“public health” OR “community health” OR “population health” OR “preventive health” OR “public well‐being” OR “health promotion” OR “public healthcare” OR “health protection” OR “public hygiene” OR “public wellness” OR “community well‐being”) AND PUBYEAR > 2018 AND PUBYEAR < 2025. The metadata of 154 articles, 66 review, 16 book chapter, 11 conference paper, 7 book, 3 editorial, 1 short survey, 1 note were initially identified.

### 2.3 Selection Criteria

All articles that explicitly addressed the topics of “microplastics” and “public health” were included. This broad inclusion allowed us to capture a variety of research and perspectives in this emerging field. However, articles that were not written in English were excluded due to potential difficulties and variations in translation that could affect the accuracy of the analysis. In addition, papers that did not provide sufficient data for scientometric analysis were excluded.

### 2.4 Procedures

Data analysis was carried out using SciVal and R Studio tools. First, a comprehensive search was performed in SciVal using keywords relevant to “microplastics” and “public health.” The search results were exported in a format compatible with Bibliometrix, a bibliometric analysis tool based on R. Once exported, the data was imported into Bibliometrix for analysis. Bibliometrix provides a comprehensive framework for the quantitative analysis of textual data in bibliometrics, allowing a detailed assessment of documentary production, collaborations between authors and countries, and thematic evolution.

### 2.5 Data Analysis

Several analyses were performed to examine document production, author–country collaborations, and thematic evolution. Bradford's law was applied to analyze the distribution of publications, and Lotka's law was applied to examine author productivity. A cross‐country collaboration map was created to visualize the interactions between countries. Thematic evolution was analyzed by identifying the focus topics over time.

## 3 Results

Several journals have demonstrated significant scholarly output and impact in 2023. The Science of the Total Environment leads with a scholarly output of 24, a CiteScore of 17.6, and a SNIP of 1.82. The Journal of Hazardous Materials follows with an output of 16, a remarkable CiteScore of 25.4, and a SNIP of 2.08. Environmental Pollution and Chemosphere also show substantial contributions with outputs of 10 and 7, CiteScores of 16 and 15.8, and SNIPs of 1.55 and 1.52, respectively. Other noteworthy journals include the International Journal of Environmental Research and Public Health, Marine Pollution Bulletin, Water (Switzerland), Environmental Research, Environmental Science and Pollution Research, and Frontiers in Microbiology (Table 1).

**Table 1:** Top 10 journals on microplastics research and public health.

Scopus source	Scholarly output	CiteScore 2023	Citations per publication	SNIP 2023
Science of the Total Environment	24	17.6	40	1.82
Journal of Hazardous Materials	16	25.4	24.6	2.08
Environmental Pollution	10	16	13.9	1.55
Chemosphere	7	15.8	108	1.52
International Journal of Environmental Research and Public Health	6	7.3	60.7	1.08
Marine Pollution Bulletin	6	10.2	37.8	1.22
Water (Switzerland)	5	5.8	22.4	1
Environmental Research	4	12.6	112.5	1.5
Environmental Science and Pollution Research	4	8.7	2.2	1.14
Frontiers in Microbiology	4	7.7	29.2	1.03

In the CiteScore quartile analysis, there was an upward trend in the number of publications in the Q1 quartile from 2019 to 2023, with a slight decrease in 2024. In 2023, the Q1 quartile had the highest number of publications with 53, followed by 37 in 2024. The Q2, Q3, and Q4 quartiles also show an increase in the number of publications over the years, although to a lesser extent than the Q1 quartile. In total, 230 publications were recorded in all quartiles from 2019 to 2024. These data reflect the increasing amount of research published in these fields over time (Table 2). This rise indicates not only a heightened academic interest but also an escalating global concern regarding the impact of microplastics on both the environment and human health. The growing diversity of authors and countries involved in these studies highlights the scientific community's recognition of the necessity for a collaborative and coordinated approach to tackle these issues. This trend is likely fueled by greater public and political awareness of microplastic pollution.

**Table 2:** Impact of scientific publications by quartile on microplastics research and public health.

CiteScore quartile	Overall	2019	2020	2021	2022	2023	2024
Q1	166	3	16	21	36	53	37
Q2	37	0	3	4	7	14	9
Q3	16	0	3	2	3	5	3
Q4	11	2	1	0	2	3	3
Total	230	5	23	27	48	75	52

In the field of research on microplastics and public health, several authors have demonstrated a significant impact. Damià À. Barceló of the University of Almeria in Spain has produced three publications with an h‐index of 149, 221.7 citations per publication and 371 views per publication. Yusof Shuaib Ibrahim of Universiti Malaysia Terengganu in Malaysia, Frank J. Kelly of King's College London in the UK, and Sabine M. Matallana‐Surget and Richard S. Quilliam of the University of Stirling in the UK have also made significant contributions. In addition, Stephanie L. Wright of Imperial College London in the UK, S. Aanand of Tamil Nadu Dr. J. Jayalalithaa Jayalithaa Fisheries University in India, Qiuying An of the University of Chinese Academy of Sciences in China, Takaomi Arai of Universiti Brunei Darussalam in Brunei Darussalam, and Tapos Kumar Chakraborty of Jashore University of Science and Technology in Bangladesh have contributed to the growing body of research in this field. These authors have demonstrated the importance of continued research on microplastics and their impact on public health (Table 3).

**Table 3:** Top 10 authors and affiliations on microplastics research and public health.

Author	Affiliation	Country	Scholarly Output	h‐index	Citations per Publication	Views per Publication
Barceló, Damià À.	University of Almeria	Spain	3	149	221.7	371
Ibrahim, Yusof Shuaib	Universiti Malaysia Terengganu	Malaysia	3	11	6.7	78.3
Kelly, Frank J.	King's College London	United Kingdom	3	87	219.3	116
Matallana‐Surget, Sabine M.	University of Stirling	United Kingdom	3	17	22.3	41.7
Quilliam, Richard S.	University of Stirling	United Kingdom	3	33	22.3	41.7
Wright, Stephanie L.	Imperial College London	United Kingdom	3	17	199	116.3
Aanand, S.	Tamil Nadu Dr. J. Jayalalithaa Fisheries University	India	2	10	13	23.5
An, Qiuying	University of Chinese Academy of Sciences	China	2	4	12	6.5
Arai, Takaomi	Universiti Brunei Darussalam	Brunei Darussalam	2	38	1	12
Chakraborty, Tapos Kumar	Jashore University of Science and Technology	Bangladesh	2	10	0.5	51

From 2019 to 2024, 156 sources and 259 documents related to microplastics and public health research were identified. The annual growth in document production was 56.87%, with an average document age of 1.65 years and an average of 28.83 citations per document. A total of 19,272 references were registered. In terms of author keywords (DE), 837 were identified. There are 1348 authors, of which 10 are authors of single‐author papers. In terms of collaboration among authors, 10 single‐authored papers were included, with an average of 5.93 coauthors per paper. The percentage of international coauthorships was 32.43%. In terms of document types, 154 articles, 7 books, 16 book chapters, 11 conference papers, 3 editorials, 1 note, 66 reviews and 1 short survey were identified. These data reflect the diversity and richness of research in this field (Table 4).

**Table 4:** Main information on microplastics research and public health.

Description	Results
Timespan	2019:2024
Sources	156
Documents	259
Annual growth %	56.87
Document average age	1.65
Average citations per doc	28.83
References	19272
Author's Keywords (DE)	837
Authors	1348
Authors of single‐authored docs	10
Single‐authored docs	10
Co‐authors per Doc	5.93
International co‐authorships %	32.43
Article	154
Book	7
Book chapter	16
Conference paper	11
Editorial	3
Note	1
Review	66
Short survey	1

**Figure 1:**
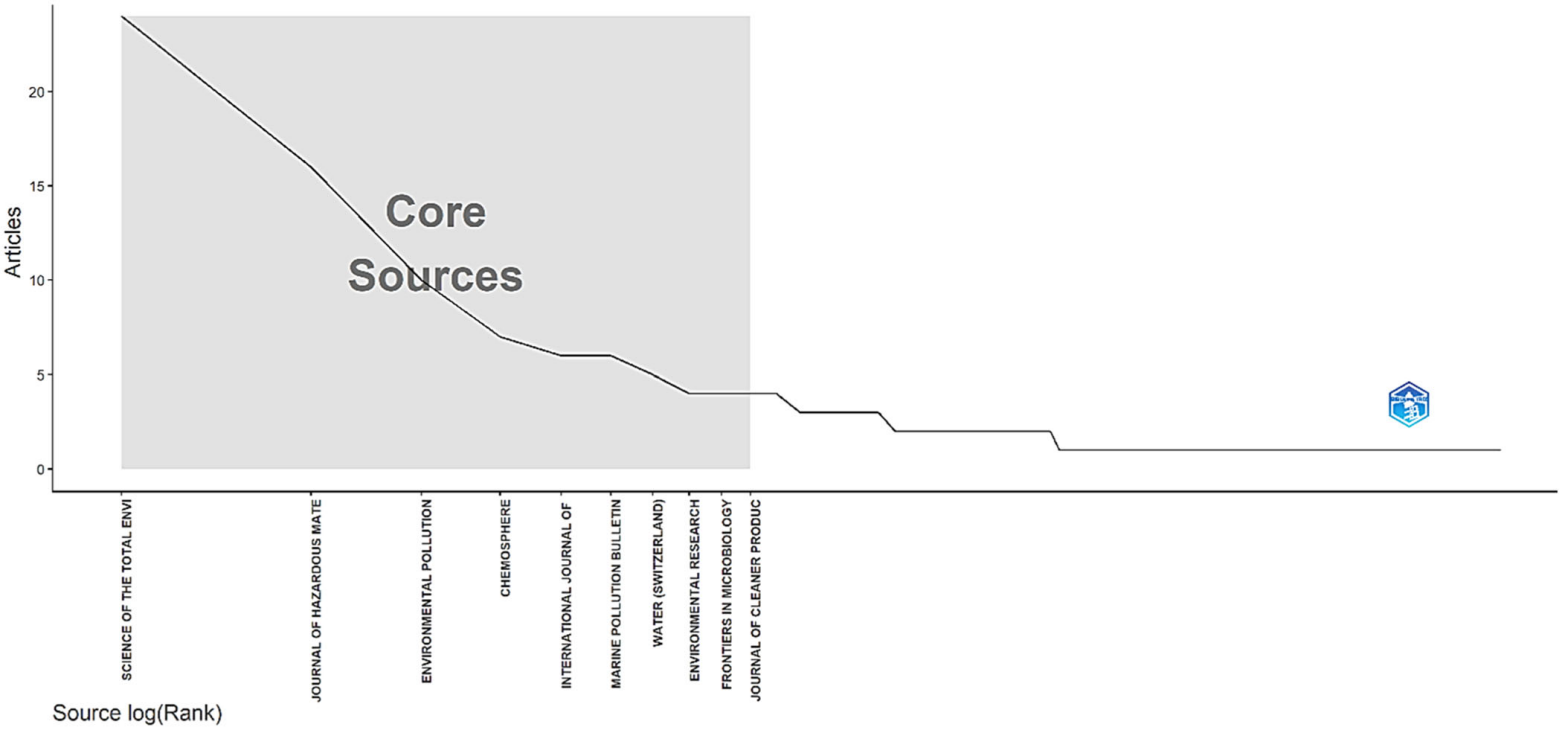
Core sources on microplastics research and public health.

According to Bradford's law, the results were divided into three zones. In Zone 1, the journals with the highest frequency of publications were “Science of the Total Environment” (24 publications) and “Journal of Hazardous Materials” (16 publications). In Zone 2, the journal “Toxics” leads with 4 publications, followed by several journals with three or two publications, such as “Chemical Engineering Journal,” “Emerging Contaminants,” “Heliyon,” among others. Finally, in Zone 3, there are many journals with only one publication, such as “Environmental Analysis Health and Toxicology,” “Environmental Fluid Mechanics,” among others (Figure 1).

**Figure 2:**
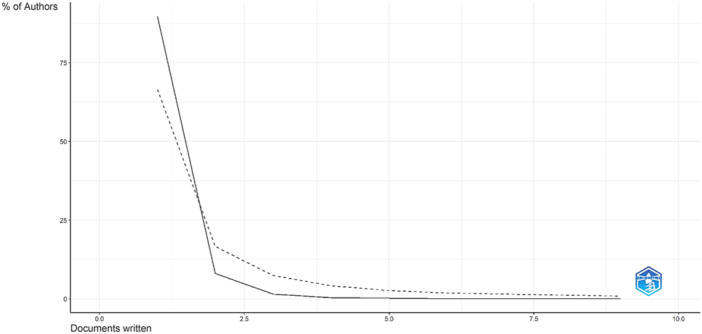
Author productivity on microplastics research and public health.

According to Lotka's law, which describes the frequency of publication by authors in a specific field, most authors (1209, representing 89.7% of the total) have written only one document. A smaller number of authors have written two papers (109 authors, 8.1% of the total), and even fewer have written three papers (20 authors, 1.5% of the total). Some authors have written four or five papers (five and three authors, respectively, representing 0.4% and 0.2% of the total). Only one author has written six papers, while another author has written nine papers, each representing 0.1% of the total number of authors (Figure 2).

**Figure 3:**
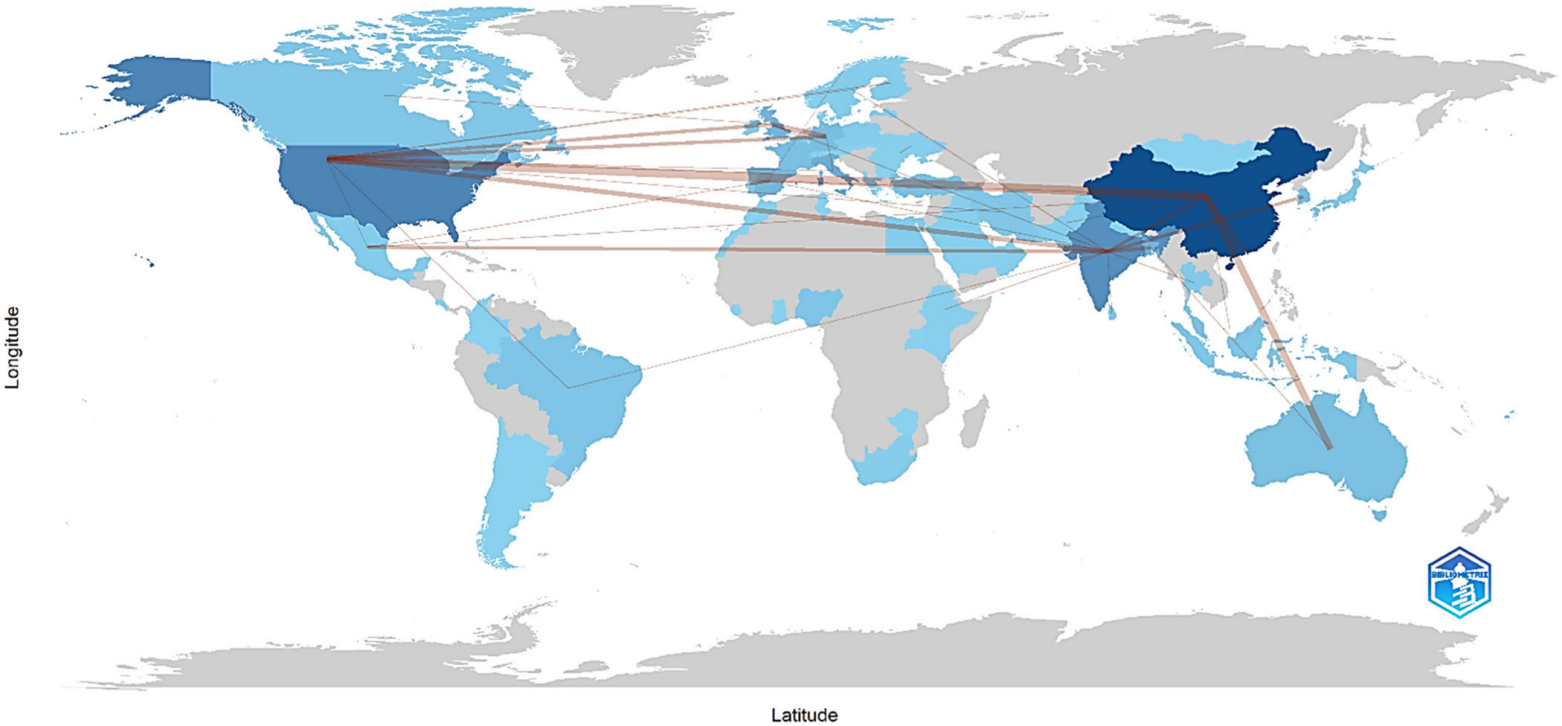
Country collaboration map on microplastics research and public health.

The cross‐country collaboration map shows the interactions between countries in terms of co‐authored publications. For example, Australia has collaborated with the Bahamas, Canada, the Czech Republic, Poland, Saudi Arabia, Singapore, and the United Arab Emirates on one publication each. Bangladesh has collaborated with Argentina, Australia (two publications), Brazil, the Czech Republic, Finland, Germany, Japan, Korea, Mexico, Nigeria, Saudi Arabia, Spain, Sweden, Thailand, and the United Kingdom in one publication each. China has had the most collaborations with Australia (six publications), Hong Kong (three publications), India (three publications), and the United States (nine publications). These data reflect the global nature of microplastics and public health research, with collaborations spanning multiple countries and continents (Figure 3).

**Figure 4:**
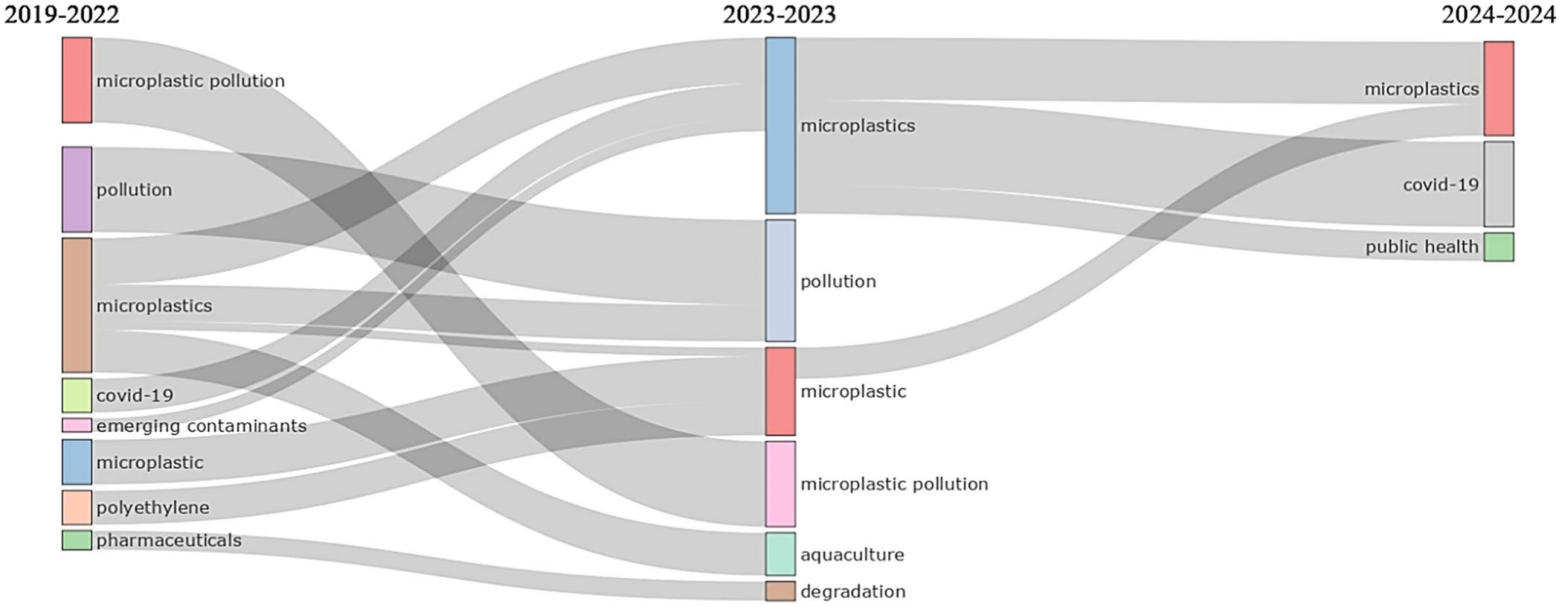
Thematic evolution on microplastics research and public health.

The thematic evolution of microplastics and public health research reveals interesting changes in focus topics over time. For example, the topic of “covid‐19” that was prominent in 2019–2022 has been linked to “microplastics” in 2023. Similarly, “emerging contaminants” in 2019–2022 has been linked to “microplastics” in 2023, with a focus on “human health.” The term “microplastics” has maintained its relevance from 2019 to 2023, as has “microplastic pollution.” The topic of “microplastics” in 2019–2022 has been expanded in 2023 to include topics such as “aquaculture” and “exposure.” In 2023, the “microplastics” topic was linked to “covid‐19” and “public health” in 2024, indicating a possible intersection of these topics in future research (Figure 4).

## 4 Discussion

The rationale for evaluating the years 2019–2024 is that during these years new environmental policies have been implemented globally, such as the EU Plastics Strategy and various national initiatives to reduce the use of single‐use plastics. In addition, societal events such as growing public awareness of plastic pollution and environmental movements have boosted research in this field. Significant publications and pioneering studies on the effects of microplastics on human health and ecosystems have also emerged in this period, providing a solid basis for trend analysis. This timeframe allows capturing the impact of these policies, events and publications, providing a comprehensive and up‐to‐date view of the evolution of microplastics research and its relevance to public health [4, 5, 6, 7, 8, 9, 10].

In recent years, microplastics have been found in various sources close to humans. Therefore, it has been reported that this new risk factor is associated with diseases, thus impacting public health [9]. This research constitutes the first study to analyze the worldwide scientific production of microplastics and public health. This analysis provides a better understanding of the magnitude of the problem and provides a basis for future research and public policy. The findings also highlight the urgent need to address this issue from a multidisciplinary perspective.

Bibliometric analyses are fundamental tools for quantifying and evaluating scientific production on specific topics. These studies allow the identification of possible co‐authors and institutions, facilitating the generation of new collaborations between researchers. In addition, they provide a detailed view of the thematic evolution in each field, which can predict the course of topics with the greatest impact. This predictive capability is essential for strategic planning in research because it allows scientists to direct their efforts toward areas with potential for high impact and emerging relevance [11]. Furthermore, these analyses can identify gaps in current knowledge, thereby suggesting opportunities for innovative studies. One significant factor is the heightened media coverage, which has elevated public awareness about the environmental and health impacts of microplastics. This increased visibility has motivated researchers to delve deeper into the subject. Additionally, regulatory changes and the introduction of new environmental policies in various countries have spurred research in this field. These policies aim to mitigate plastic pollution and encourage sustainable practices [9, 11, 12, 13, 14].

Our results indicate that the Journal Science of the Total Environment has the highest scientific output among the areas studied. This finding is consistent with other bibliometric analyses related to emerging pollutants and organophosphates, in which this journal is positioned as one of the most prolific [12, 13]. On the other hand, Environmental Research stands out as the journal with the highest impact, which is consistent with previous analyses on the association between environmental toxins and preterm delivery [14]. In addition, the articles with the highest production have a similar thematic distribution, focusing on pollution and its health risks [12]. Note that more than half of the articles were published in first‐quartile journals, demonstrating the great interest and impact of the scientific community on this topic.

When evaluating the period 2019–2024, Damià À. Barceló emerged as the most prolific author with the highest impact. The article with the highest number of citations was a review that addressed the increase in microplastic contamination during the COVID‐19 pandemic. In this article, recommendations, such as implementing improved plastic waste management systems and promoting the use of bioplastic, are provided [15]. Another article with many citations addressed the impact of microplastic pollution on enzyme activity and bacterial community structures [16]. Note that four of the ten most productive authors are from the United Kingdom. This finding is consistent with a bibliometric analysis of microplastic pollution in marine environments, in which the UK is positioned as a major contributor to research in this area [17].

Our plot of Lotka's law shows that most authors have published only one article, whereas a small group of authors contributed multiple publications. These results suggest the need for a more inclusive approach to scientific productivity assessment that recognizes both prolific researchers and those who contribute sporadically. In addition, the Chinese authors demonstrated strong collaboration with colleagues from the United States and Australia, which is consistent with a bibliometric analysis on another relevant public health topic [18]. These findings suggest that these countries have established strong collaborative networks around a specific topic.

During the period 2019–2022, microplastics research was closely related to the COVID‐19 pandemic, reflecting the onset of the global health crisis. In the second stage, the focus shifted to the impact of microplastics in aquaculture, due to growing evidence that these contaminants affect marine species at the molecular and cellular levels, as they are found in their diets and the environment [19]. This growing concern led researchers in the last period to address the impact of microplastics on public health, highlighting their relevance in epidemiology.

Our study has some limitations that should be considered before interpreting the results. First, the search and extraction of articles were performed exclusively in the Scopus database, which may have excluded articles published in other databases. However, Scopus is one of the most extensive and rigorous databases in terms of publication quality [20]. Second, the selection of articles was limited to a specific period, excluding articles published before 2019. However, it is worth mentioning that less than 10 articles were published in that period and that a high‐impact study was published in 2018, the effects of which were mainly observed from 2019, marking a turning point for a significant increase in scientific production [21, 22]. Finally, when implementing a search strategy, there is the possibility of selecting false positives. However, our study included rigorous selection criteria before conducting the analysis, which minimized this risk.

Despite the significant advancements in microplastic research, there are several limitations that need to be addressed. One major limitation is the lack of standardized methods for sampling, analyzing, and reporting microplastic data, which hampers the comparability of studies. Future research should prioritize the development of standardized protocols, expand the scope to include various environmental compartments, and investigate the chronic impacts of microplastics on human health. Enhanced interdisciplinary collaboration and increased funding are essential to address these gaps and advance our understanding of microplastic pollution and its implications for public health.

The relevance of the present study that brings several novelties compared to previous research on microplastics and public health [23, 24, 25, 26]. First, it uses a scientometric approach to analyze the spatio‐temporal trends and dynamics of the scientific literature, providing a broader and quantitative view of the development of microplastics research. Furthermore, it covers a recent and significant period (2019–2024), highlighting the growth in article production and international collaboration, which is crucial to understand the evolution of global interest and cooperation on this topic. The research also highlights the thematic evolution, showing how microplastics have been linked to emerging issues such as COVID‐19 and public health, underlining the contemporary relevance of the problem. These features make our study not only document the current state of microplastics research, but also provide a unique perspective on its evolution and future trends in public health.

## 5 Conclusion

This study provides a comprehensive analysis of microplastics and public health research from 2019 to 2024. Over this period, there has been a notable increase in the number of published papers, accompanied by a rise in international collaborations and a variety of publication types. The study identifies the most prolific authors and journals, underscoring the critical need for ongoing research in this area. Insights from Bradford's and Lotka's laws reveal patterns in publication distribution and author productivity. The global nature of this research is highlighted by a cross‐country collaboration map, showing extensive international partnerships. Thematic evolution analysis indicates significant shifts in research focus, particularly the intersection of microplastics with topics such as COVID‐19 and public health. These findings emphasize the importance and urgency of continued research on microplastics and public health, pointing to promising future research directions.

## Author Contributions

**Juan Alvitez:** conceptualization, investigation, writing–original draft. **Luis Huarachi:** conceptualization, investigation, writing–original draft. **Abigail Temoche:** conceptualization, investigation, writing–original draft. **Carlos Medina:** conceptualization, investigation, writing–original draft. **Daniel Alvitez‐Temoche:** conceptualization, investigation, writing–original draft. **Miguel Cabanillas‐Lazo:** writing–original draft, methodology. **Frank Mayta‐Tovalino:** conceptualization, writing–review and editing, software, data curation, supervision.

## Conflicts of Interest

The authors declare no conflicts of interest.

## Data Availability

The data that support the findings of this study are available from the corresponding author upon reasonable request.
